# The absorption and uptake of recombinant human follicle-stimulating hormone through vaginal subcutaneous injections - a pharmacokinetic study

**DOI:** 10.1186/1477-7827-7-107

**Published:** 2009-10-07

**Authors:** Chao-Chin Hsu, Hsin-Chih Kuo, Chao-Tien Hsu, Qing Gu

**Affiliations:** 1 Department of Obstetrics & Gynecology, National Cheng Kung University Hospital, Tainan, 701, Taiwan; 2 Department of Obstetrics & Gynecology, China Medical University, 91 Hsueh-Shih Rd, Tai-Chung, 404, Taiwan; 3 Department of Health Management, I-Shou University, Kaohsiung, Taiwan; 4 Department of Pathology, E-Da Hospital and I-Shou University, Kaohsiung, Taiwan; 5 Cambridge Bay Assisted Conception Unit, Chang Hai Hospital, Shanghai, China

## Abstract

**Background:**

Follicle stimulating hormone (FSH) has been routinely used for ovulation induction. Because of rapid clearance of the hormone, FSH is commonly administered by daily intramuscular or subcutaneous injections in in-vitro fertilization (IVF). To reduce the number of visits to the clinic, an intermittent vaginal injection of rhFSH every 3 days employing the concepts of mesotherapy and uterine first-pass effect was invented and has successfully been applied in women receiving IVF treatment. This study was designed to monitor the pharmacokinetic pattern of rhFSH administered vaginally.

**Methods:**

Twelve healthy women with regular ovulatory cycles were recruited. All volunteers received gonadotrophin-releasing hormone agonist to suppress pituitary function and were assigned to receive single dose recombinant human FSH (rhFSH, Puregon 300) either using conventional abdominal subcutaneous injection or vaginal subcutaneous injection in a randomized cross-over study. Serum samples were collected at pre- scheduled time intervals after injections of rhFSH to determine immunoreactive FSH levels. Pharmacokinetic parameters characterizing rate [maximal plasma concentrations (Cmax) and time of maximal plasma concentrations (tmax)] and extent [area under the plasma concentration-time curve (AUC) and clearance] of absorption of rhFSH were compared.

**Results:**

Vaginal injection of rhFSH was well tolerated and no drug-related adverse reaction was noted. Our analysis revealed that tmax was significantly earlier (mean 6.67 versus 13.33 hours) and Cmax was significantly higher (mean 17.77 versus 13.96 IU/L) in vaginal versus abdominal injections. The AUC_0-∞_ was 1640 versus 1134 IU·hour/L in vaginal and abdominal injections, respectively. Smaller plasma elimination rate constant (0.011 versus 0.016 hour-1), longer mean residence time (106.58 versus 70.47 hours), and slower total body clearance (292.2 versus 400.1 mL/hour) were also found in vaginal injection.

**Conclusion:**

The vaginal injection mode elicited a rapid and highly extended absorption of rhFSH injected compared to conventional abdominal injection. These data indicate that the rate and extent of FSH absorption from the injection site can vary depending on the route of the FSH administration.

## Background

Follicle stimulating hormone (FSH) has been widely employed for ovulation induction and in-vitro fertilization. The ability of FSH to promote folliculogenesis is dependent on the plasma level of FSH that develops after administration [1-3]. The relatively short elimination half-life and rapid metabolic clearance of current FSH preparations requires that daily injections are administered to maintain steady state FSH levels above the threshold level during ovarian stimulation [4]. The invention of recombinant human FSH (rhFSH) preparations, devoid of other gonadotrophins or inactive contaminants, made self-subcutaneous-injection possible [5]: commonly, the patient's spouse or another non-professional individual will perform the injections. However, in under-developed and developing countries or areas such as in Taiwan and China, many women still travel every day to receive gonadotrophin injections at the IVF clinic [6].

To reduce the number of visits to the clinic, an alternative treatment regime of vaginal injections of rhFSH every 3 days [7] employing the concepts of mesotherapy [8] and uterine first-pass effect [9,10] has been developed. Mesotherapy [11], the injection of minimal amounts of medicines into the hypodermis and dermal layer at weekly intervals, can achieve the same pharmacological effects as daily intramuscular injections. The basic premise of mesotherapy was: smallest dose, infrequently, in the correct location [8]. A countercurrent exchange with vein-to-artery diffusion between upper vagina and uterus has been shown to result in the uterine first-pass effect [9,10]. This fact has been applied in the vaginal administration of progesterone supplements, with much higher progesterone delivery to the uterus when compared with intramuscular injections [12]. The existence of the uterine-ovarian countercurrent system has also been proven [13].

Intermittent vaginal injection of rhFSH has successfully been applied in women receiving IVF treatment [14]. The present study was a follow up of our previous works and designed to monitor the pharmacokinetic pattern of rhFSH administered vaginally versus abdominally and to investigate whether plasma level of FSH differs depending on the route of the hormone administration.

## Methods

### Study population

Twelve healthy women with regular ovulatory cycles were recruited. They were in good cooperative condition and had not been exposed to oral pills or other forms of hormone therapy for at least 3 months preceding the study. All volunteers were nonsmokers, ovulatory (with a mean cycle length of 26-32 days and intra-subject variation of ± 2 days), and without evidence of ovarian disease. The uterine cervix Pap smears for all volunteers were normal. Women with evidence of polycystic ovarian syndrome or ultrasound evidence of ovarian cysts were excluded from the study. All subjects were also devoid of any medical disorders such as hypertension, hepatic or renal diseases, and endocrine abnormalities. All subjects signed the informed consent to the study protocol, which had been approved by the Ethical Committee of the institution. The study was conducted in agreement with the Declaration of Helsinki for Medical Research Involving Human Subjects.

### Study design

This was a randomized, single-center, two-period cross-over study, with each subject undergoing two trial cycles using alternative injection sites. The site for the first cycle of injection was randomized to be either the abdomen or vagina. All subjects received oral pills (Marvelon, containing 0.030 mg ethinyl oestradiol and 0.150 mg desogestrel, NV Organon, Oss, The Netherlands), starting at day 3 of the precedent cycle. A gonadotrophin-releasing hormone (GnRH)-agonist nasal spray (Buserelin acetate, Aventis Pharma Deutschland GMBH, Frankfurt, Germany) was given daily from day 18 of that cycle to achieve proper pituitary down-regulation of GnRH followed ovarian hyperstimulation [14] and GnRH-agonist was maintained throughout ovarian hyperstimulation to the last day of the monitoring in each mode of injection. On the morning of period day 3, the study subjects visited the clinic where a 20-gauge intravenous catheter with a heparin lock was placed at forearm and levels of baseline plasma hormones including FSH, estradiol (E_2_) and lutenizing hormone (LH) were monitored. A vaginal ultrasonographic scan was performed to check for the absence of ovarian cyst or any ovarian pathology. The subjects then received rhFSH injections in one of two injection modes once pituitary suppression was achieved as manifest by serum E_2 _< 50 pg/mL, LH < 2.5 mIU/mL, and FSH < 10 mIU/mL. All subjects received a single dose of Puregon solution for injection in cartridges [rhFSH, Puregon 300, Organon (Ireland) Ltd. Swords, Co. Dublin, Ireland]. The formulation was a ready-for-use solution containing 833 IU/mL in-vivo bioactivity of FSH.

For vaginal injections, the subjects were placed in Trendelenburg's position after ultrasonographic scanning. A sterilized speculum was inserted and vaginal discharge thoroughly cleaned with sterilized normal saline and Povidone iodine solution. The rhFSH (one vial of Puregon 300, ~425 IU rhFSH in 0.525 mL) was aspirated into a 1 mL syringe fitted with a 30 gauge × 1/2 inch (0.31 × 13 mm) needle. To mimic the serum concentrations of rhFSH administered in clinical IVF protocol, the injection was performed as that used in ovarian hyperstimulation [14] with the needle angled at 15-30° towards the vaginal mucosa and inserted at the middle to upper portions of bilateral vaginal wall at positions corresponding approximately to 3 o'clock and 9 o'clock. The rhFSH was injected at a depth of 1-2 mm into the vaginal wall. For abdominal injections, rhFSH solution was injected with a 26 gauge × 1/2 inch (0.45 × 13 mm) needle subcutaneously at a depth of 13 mm under the skin of anterior abdominal wall at the level of the umbilicus 5-10 cm from the midline.

The procedure was repeated in a two-way random sequence, cross-over fashion: once a cycle with one mode of injection was completed, for volunteers' convenience, the subjects were monitored for 2-6 months before the second cycle with the alternate mode of injection was carried out. The time frame of 2-6 months provides a sufficient washout to prevent the carry-over effects and this also fits well with clinical practice that a successive IVF cycle to be carried out. After vaginal rhFSH injections, the subjects were asked to keep abstinence for at least one week to avoid the interference of vaginal blood flow alterations during intercourse. Subjects were allowed to resume their normal activities after the injection.

### Assessment

In addition to baseline samples, peripheral blood samples were taken at time intervals after the injections as below: 0.5 (optional), 1, 2, 4, 6, 8, 10, 12, 14 (optional), 24, 48, 72, 96, 120 hours, and optionally 144, 168, 192, 216, 240, 288, 312, 336, 360 hours. Exudates from the injection points in the vaginal cavity immediately after vaginal injections were collected. Serum samples and supernatant of vaginal exudates were frozen at - 20°C until assessment. All hormones were measured using commercially available kits (Diagnostic Systems Laboratories, Inc. Webster, Texas, USA). Estradiol concentrations were measured by a competitive radioimmunoassay (Ultra-sensitive estradiol RIA DSL-4800). The sensitivity of the assays was 2.2 pg/mL and the inter-assay coefficient of variation (CV) was 8.0%. Follicle-stimulating hormone and LH concentrations were measured by a two-site immunoradiometric assay (FSH IRMA DSL-4700, LH IRMA DSL-4600) with two monoclonal antibodies. Data are expressed in terms of IRP 78/549 and 68/40 for FSH and LH, respectively. The sensitivity of the assays was 0.11 mIU/mL and 0.12 mIU/mL and the inter-assay CV was 6.2% and 7.0%, respectively for FSH and LH. Follicle growth was followed up at 72 hours and 120 hours after injections.

### Pharmacokinetic analysis

The data from the plasma concentration versus time curve were evaluated to derive the following pharmacokinetic parameters: measured maximal plasma concentration (C_max_); time to reach measured maximal plasma concentration (t_max_); plasma elimination rate constant (K_el_), determined from simple linear regression based on the terminal log-linear part of the last quantifiable plasma concentration versus time profile; apparent half-life (t_1/2_), the half life determined with the terminal phase of the last quantifiable plasma concentration-time profile, calculated as t_1/2 _= (0.693)/K_el_; summation of area under the plasma concentration-time curve from time zero to the time of last quantifiable concentration by the linear trapezoidal rule (AUC_0-t_); summation of area under the plasma concentration-time curve from time zero to the time of last quantifiable concentration (AUC_0-∞_), calculated by the linear trapezoidal rule, and extrapolated to infinity estimated by the last quantifiable concentration divided by K_el_; mean residence time (MRT)_; _square of the correlation coefficient between the observed and modelled data values (RSQ), indicates the proportion of variability in a data set that is accounted for by a statistical model;volume of distribution based on the terminal phase (Vz); and total body clearance (CL = Dose/AUC). All pharmacokinetic parameters were calculated using non-compartment methods with the computer program WinNonlin™, version 5.2 (Scientific Consulting Inc., Apex, NC, USA). In this instance, the endogenous serum FSH concentration (baseline concentration) was subtracted from all after dosing serum FSH concentration, thereby, assuming a constant endogenous serum FSH over the study interval [15].

### Statistical analysis

The comparison of the rate of absorption for the two injection modes was tested using C_max_, and t_max_, while the extent of absorption was tested using AUC_0-∞_, AUC_0-t_, and CL. The conventional abdominal injection was taken as reference formulation and the vaginal injection as test treatment. All pharmacokinetic parameters were calculated at 95% confidence intervals. Comparison of pharmacokinetic parameters, between the phases of administration, was performed using paired tests (Minitab software, version 14). A one-way analysis of variance (ANOVA) was performed for the comparison of mean values of pharmacokinetic parameters between two injection modes. We also used MANOVA (SPSS, version 12.0) for repeated measures to ascertain differences (within-subjects and between-subjects effects) with respect to the changes of FSH concentration in the time sequence of this trial. A post hoc multiple comparisons on both injection modes was performed using Dunnett (2-sided) t test.

Data are presented as mean ± SD unless stated otherwise. Differences were considered to be statistically significant if P < 0.05. As this administration method was a new invention and there was no previous information regarding the rate and extent of FSH absorption employing this injection method, we did not carry out the power analysis a priori. For number of volunteers (12 cases) recruited, we followed the pioneer works on the pharmacokinetics of recombinant human FSH (15).

### Safety parameters

The injection sites were monitored at 1, 24 and 72 hours post injection for redness, itching, swelling, pain and bruising with the scoring as none, mild, moderate or severe. Any abnormal discharge or lesions during pelvic examination or vaginal injection was reported.

### Ethical Consideration

Informed consent was given. All subjects were told exactly why this study was taking place and the potential benefits from the study, and then invited to take part without any pressure being put on them to do so. Subjects were allowed to withdraw freely at any time, and if they did, their contribution to the study removed. The researcher did as much as possible to protect subjects from any harm resulting from their participation in this study, and to minimize disturbances to the subjects. The records of individual subjects were kept confidential and any information that will be published from the study will not identify the result of specific individuals.

## Results

The mean age of the twelve subjects was 31.2 ± 7.3 (range 20-43) years with mean body weights of 46.1 ± 3.4 (range 39.4-50) kg, mean body height of 156 ± 5 (range 150-165) cm, and mean body mass index of 19.4 ± 2.1 (range 17.3-24.4) kg/m^2^. All subjects completed two cycles of rhFSH injections. Immediately before administration of rhFSH, the mean plasma FSH concentration was 5.16 ± 2.16 IU/L versus 5.45 ± 2.32 IU/L and the mean plasma E_2 _concentration was 25.4 ± 20.9 pg/mL versus 25.3 ± 17.0 pg/mL for abdominal and vaginal injections, respectively. After rhFSH injections, blood samples were taken as scheduled up to 120 hours in all subjects. In six subjects receiving vaginal injections and two receiving abdominal injections, further blood samples were taken up to 360 hours after injection. In vaginal injections, 0.5-3 mL of bloody exudates was aspirated immediately after injections. The volumes of exudates were relatively consistent among injections with median value of 0.7 mL and only one case had exudates over 1 mL. The mean FSH concentration in the exudates was 30.75 ± 7.54 IU/L. Thus, of the amount of rhFSH was lost during the injection procedure was only small portion of rhFSH (425 IU) injected. No exudates were noted after abdominal injections.

### Local tolerance

Local reactions were all mild and generally short-lived in both administration modes. After abdominal injections, redness, swelling and pain were reported in one subject at 1 hour but none at 24 and 72 hours. Bruising occurred in two subjects at 1 hour and persisted at 24 and 72 hours. After vaginal injections, no redness or swelling was found at 1 and 24 hours. Pain was experienced by two subjects during vaginal injection but none at 1 and 24 hours. Bruising occurred in three subjects at 1 hour and in two subjects persisted until 24 and 72 hours. No subjects experienced an itching sensation in either injection modes.

### Pharmacokinetic analysis

**Figure 1:**
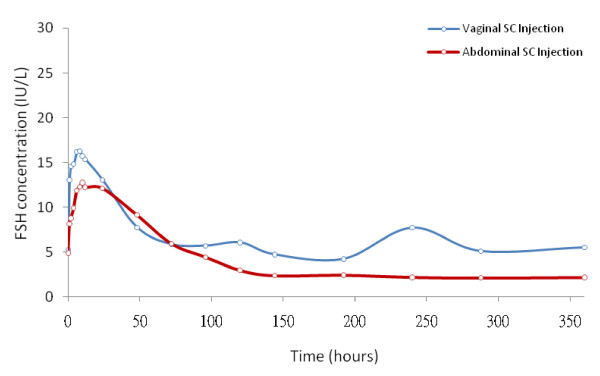
**Plasma FSH concentrations after abdominal or vaginal subcutaneous injections of 425 IU recombinant human FSH in twelve women under pituitary down-regulation with GnRH-agonist**. Mean FSH concentrations versus time points were presented. The vaginal injection mode elicited a rapid and higher extended absorption of FSH injected.

**Figure 2:**
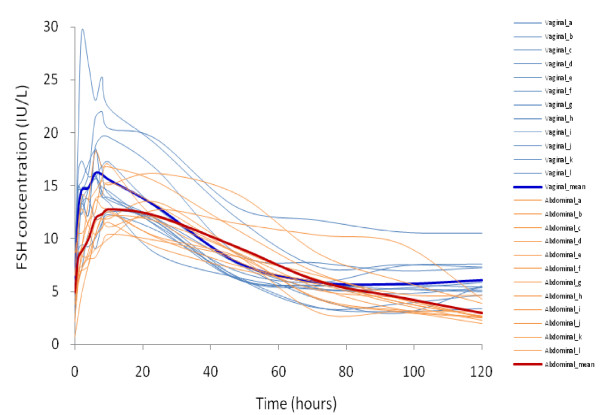
**Plasma concentration-time profile of each individual and mean increases of immunoreactive FSH after abdominal and vaginal subcutaneous injections of 425 IU recombinant human FSH in twelve women under pituitary down-regulation with GnRH-agonist**. Compared to baseline FSH level, significant differences were noted at 1 to 24 hours of vaginal injections and at 1 to 48 hours of abdominal injections (Table 5 & 6, See Additional files 5 &6).

The mean data of plasma FSH at different time intervals are shown in Figure 1. There is moderate to high inter-individual variability; the mean CV for vaginal injection is 32% and for abdominal injection is 27% for raw data calculated up to 120 hours. In general, the plasma FSH levels could be divided into 3 phases: 0-24 hours, 24-72 hours, and 72-360 hours, with higher elevated FSH concentrations in 0-24 hours in women receiving vaginal injections compared to those in women receiving abdominal injections, while in the 24-72 hours period a similar curve of FSH levels were produced in both groups with higher FSH concentrations in women receiving abdominal injections (Figure 1 and figure 2). The FSH concentrations remained at a low level (< 3 IU/L) at the time between 72 and 360 hours in those receiving abdominal rhFSH injections, but it remained on higher concentrations (> 5 IU/L) during this time-frame in those receiving vaginal rhFSH injections (Figure 1).

Only data up to 120 hours were analyzed for pharmacokinetic parameters, as not all subjects were monitored after this time point. Individual pharmacokinetic parameters are presented in Tables 1 & 2 (See Additional files 1 &2). The mean values of the pharmacokinetic parameters obtained are presented in Table 3 (See Additional file 3). After single dose of 425 IU rhFSH, peak plasma FSH concentrations occurred significantly earlier (t_max _6.67 versus 13.33 hours; P < 0.05) and were higher (C_max _17.77 versus 13.96 IU/L; P < 0.05) in vaginal versus abdominal injections. There was also increased net absorption (AUC_0-∞ _1640 versus 1134 IU·hours/L; P < 0.05) in vaginal versus abdominal injection. However the net absorption up until the last quantifiable concentration (AUC_0-t_) showed no difference in both groups. The apparent half-life, t_1/2_, was about 1.5 times longer (66 versus 46 hours; P < 0.01) in those receiving vaginal injections and this was closely associated with a significant smaller plasma elimination rate constant, K_el _(0.011 versus 0.016 hour^-1^; P < 0.005), longer mean residence time (106.58 versus 70.47 hours; P < 0.001), and a much slower total body clearance, CL (292.2 versus 400.1 mL/hour; P = 0.005) compared to those of abdominal injection. There was no difference in the volume of distribution in both modes of administration.

MANOVA for repeated measures showed significant difference of serum FSH levels in relevant to the time effect for both abdominal and vaginal injection modes (Table 4, See Additional file 4). The tests of within-subjects effects and between-subjects effects were remarkable (P < 0.01). The post hoc analysis indicated that the serum FSH concentrations showed significant difference from baseline level at 1 to 24 hours after rhFSH administration in the vaginal injection group while in the abdominal injection group the significant different values were from 1 to 48 hours after rhFSH administration (Table 5 & 6, See Additional files 5 &6).

**Figure 3:**
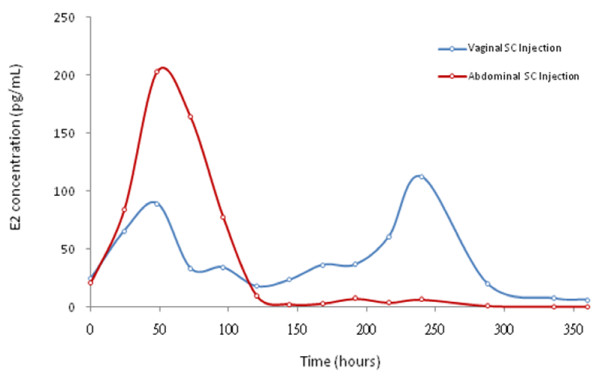
**Serum E2 concentrations after vaginal or abdominal subcutaneous injections of 425 IU recombinant human FSH in women under pituitary down-regulation with GnRH-agonist**. Mean concentrations of E2 versus time points were presented. An initial higher E2 levels were noted in those of abdominal injections but a more consistent elevated E2 was found in those of vaginal injections.

The LH level was sustained in profoundly suppressed values (< 2.0 IU/L) monitored up to 360 hours after rhFSH injections in both groups. The E_2 _level was elevated 24-96 hours after injections in both groups with higher levels noted in the abdominal injections (Figure 3). The peak level of E_2 _was 202 and 89 pg/mL at 48 hours in abdominal and vaginal injections, respectively. Following abdominal injections, the E2 concentrations dropped sharply to below 10 pg/mL after 120 hours and remained at very low level up to 360 hours. The E2 levels in those who received vaginal injections were maintained at above 30 pg/mL after 120 hours with a second peak concentration of 112 pg/mL at 240 hours and then dropped sharply to below 10 pg/mL. This pattern of E_2 _levels correlated with FSH concentrations which plateau after 72 hours with a second peak at 240 hours in the vaginal injection group (see Figure 1). No follicle larger than 13 mm was detectable at 72 hours and 120 hours after injections in both injection modes.

## Discussion

The present study defines the pharmacokinetics of rhFSH administered vaginally compared with conventional abdominal SC injection. The vaginal injection mode elicited a rapid and highly extended absorption of rhFSH injected compared to conventional abdominal injection. The enhanced absorption was indicated by a shorter time to peak plasma concentrations with higher maximal plasma concentrations, while extended absorption was manifest by higher area under the plasma concentration-time graph (AUC_0-∞_), lower elimination rate constant, and slower total body clearance. The data we obtained indicate that the rate and extent of FSH absorption from the injection site can vary depending on routes and methods of FSH administration. We have also demonstrated that vaginal SC administration of rhFSH had no adverse impact on women receiving the injection.

One of the main findings of this study is that the absorption of rhFSH injected vaginally (manifest as higher C_max _17.77 IU/L with shorter t_max _6.67 hour) was more rapid than conventional abdominal SC injections. Different pharmacokinetic data were found in studies in pituitary down-regulated normal women with C_max _of 3 to 6.9 IU/L, t_max _of 16-17 hours in SC single dose injections of 300 IU rhFSH [15-18]. High intra-individual variability and broad absorption peaks for rhFSH have been indicated to account for variations noted in the t_max _[19]. The reagents used in the pituitary down-regulation can be used to explain the variations of C_max_. The baseline FSH was suppressed to below 0.25 IU/L in cases that received high dose oral pills [19] or received GnRH-agonist depot preparations [15-17] versus below 10 IU/L in those received GnRH-agonist nasal spray [20].

The increased absorption of rhFSH is another prominent feature of the vaginal injection. Compared to previous similar studies using a single 300 IU rhFSH injection, the extent of absorption revealed by the AUC_0-∞ _in the present study (1134 and 1640 IU·hours/L in abdominal and vaginal SC injections, respectively) is much higher than that of 339 IU·hours/L in IM injection [21], of 456 IU·hours/L in SC injection [18], and of 598 IU·hours/L in IV injection [15]. The higher AUC_0-∞ _in vaginal injection may lead to the impression of higher bioavailability of rhFSH administered in this mode. However, very similar relative bioavailability of 1.127 and 1.068, calculated using the formula of CL·AUC/Dose [22] were found in vaginal and abdominal injections, respectively. Thus, the correct dose (425 IU in this study) of rhFSH was injected in both modes to achieve a therapeutic systemic exposure. A more comparable pattern of AUC_0-t _in this study further reflected similar relative bioavailability of both injection modes. The total clearance of 0.40 and 0.29 L/hour in abdominal and vaginal SC injections, respectively in this study is much slower than a previous study of 0.5-0.6 L/hour in IV injection of 300 IU of rhFSH [15] and of 0.75 L/hour in SC injection of 150 IU of rhFSH [19]. The volume of distribution was around 3 L in both groups, indicating that the rhFSH was generally distributed in plasma. The elimination rate constants in the present study (0.016 versus 0.011 hours^-1 ^in vaginal versus abdominal injections) are also much lower than that of 0.03 hours^-1 ^in both SC and IM injections of HMG [20]. The mean residence time of 107 hours in vaginal injection versus 70 hours in abdominal injection is well correlated with longer apparent plasma half-life.

Though the endogenous FSH was not fully suppressed in the present study, the estimation of elimination half-life of 46 hours in abdominal SC injection of the present study is similar to that of 37 hours in SC/IM [16,17], and 44 hours in IM [21] single 300 IU rhFSH injections. However, the estimation of apparent plasma half-life (66 hours) for vaginal injection in the present study is about 1.5 times of that for abdominal injection. We recognize that there may present minimal endogenous FSH secretion in this study. The elimination half-life of FSH obtained from cases received abdominal SC injections was very similar to those of previous studies employing fully suppressed pituitary endogenous FSH secretion. Thus, the lower level fluctuation of baseline endogenous FSH secretion may not affect the result of pharmacokinetic analysis in the present study. As this is a cross-over study, volunteers received both abdominal and vaginal injections, FSH concentrations in the vaginal injection group are thus comparable to those of abdominal injection. A similar pattern of prolonged elimination half-life could only be found in studies using long-acting FSH preparations with either a C-terminal modified form of FSH resulting in slow absorption [23,24] or the glycosylation of an N-terminal extension to prolong the half-life of FSH [25]. Chemical alterations of FSH, such as the removal of sugars from the carbohydrate side chains or nicks in the peptide chains, at injection sites before absorption into general circulation, has been noted to affect the mean residence time and the rate of clearance [20]. The possibility that vaginal injection may lead to alteration of peptide chains of rhFSH at injection sites or affect the galactose-terminal complex of rhFSH and its clearance rate and plasma half-life needs to be investigated further.

Studies have shown that some drugs administered vaginally may provide rapid and complete systemic absorption and much higher levels of both E_2 _and estrone were found following the vaginal dose compared to the oral dose [26]. The development of vaginal progesterone administration for luteal support [10,12] showed that lipid soluble steroid hormones are rapidly absorbed in the vagina, achieve quick local effects to the uterus, and avoid the first pass hepatic metabolic effect [12]. With an injectable recombinant protein such as rhFSH, though there is no identifiable mechanism to suggest that vaginal administration would be superior as is seen with steroid hormones vaginal administration, the clinical effectiveness of vaginal administration of rhFSH has been proved in our previous study [14]. Employing the concept of uterine first-pass effect and the uterine-ovarian countercurrent system, the medicines were injected into bilateral vaginal walls in our study to allow rhFSH to be transported to bilateral ovaries in a shortest distance. The injection depth we used in this study was 1-2 mm (as commonly used in the mesotherapy) into vaginal mucosa, in the areas of lamina propria and tunica propria, the connective tissue layers containing extensive vascular channels and elastic tissue [27]. A portion of rhFSH injected vaginally might have absorbed and transported more efficiently to the systemic circulation through the extensive vascular channels in these area of the vagina. This mechanism may explain the more rapid absorption of vaginal SC injection compared to abdominal SC injection. We did not have data for a single site vaginal injection, nor did we have data for two injection sites in abdominal injections. Further studies are required to clarify this issue. As far as the sexual intercourse after the vaginal injections of rhFSH might affect the distribution of medicines injected and the pharmacokinetic patterns, volunteers receiving vaginal injections were advised to abstain for at least one week. In the clinical practice of IVF treatment, the couples were commonly discouraged to have intercourse for at least 3-5 days after embryo transfer. In the present study, we do not have any data or information to support the observed pharmacokinetics in relevant to the coital activity in the peri-ovulatory period.

The concept of mesotherapy may explain why medicines persist for longer when injected into superficial subcutaneous and/or dermal layers, as was used for the vaginal injections in this study. Studies have suggested that the skin is a natural time-release system, with medicine injected into superficial subcutaneous and/or dermal layers remains in the injected area longer as it is cleared more slowly by the general circulation than medicine injected more deeply [28,29]. The anatomic characteristics of the dermal layer, with oriented tropocollagen polypeptide macromolecules and subdermal tissues merging with the fat-containing subcutaneous tissue, contribute to this effect: the skin epithelium acts as the reservoir and medicine injected into these layers may be temporarily reserved locally [30]. The vaginal epithelium and lamina propria/tunica propria might also serve as a reservoir, with medicines administered here kept within this local area and released or absorbed slowly into blood circulation. Besides the rapid absorption as described above, another characteristic of the vaginal injection mode observed in this study is the extended and prolonged smoother temporal profile of plasma FSH levels. This modulated temporal profile is likely to produce more sustained and less fluctuating plasma FSH levels during rhFSH administration. Thus, the rhFSH administered vaginally employing mesotherapy presented with two phases of absorption: a fast phase indicated by the higher C_max _with shorter t_max_, and a second phase with extend absorption reflected by the higher AUC_0-∞_, and slower total body clearance. The clinical significance of higher and more stable FSH levels for ovulation induction and IVF treatment, however, remains to be determined.

**Figure 4:**
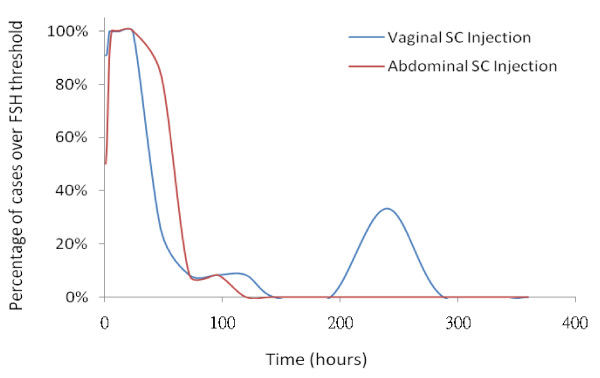
**The percentage of cases with plasma FSH level higher than the FSH threshold (> 7.8 IU/L) after abdominal or vaginal subcutaneous injections of 425 IU recombinant human FSH in women under pituitary down-regulation with GnRH-agonist**.

As the present study employed a single dose injection of rhFSH, the serum FSH level elaborated may not sustain enough to override the window of threshold for proper follicular growth and there were only transiently elevated levels of E2 detected in both groups. The serum FSH threshold for follicular growth has been found stably at approximately 7.8 IU/L in women with regular menstrual cycles [31]. Higher E2 level found in those receiving abdominal injections in this study may reflect the slightly higher serum FSH concentration (over the FSH threshold of 7.8 IU/L) up to 48 hours (Figure 2 and Table 5 & 6, See Additional files 5 &6). On the contrary, serum FSH concentration in those of vaginal injection to surpass the FSH threshold was found only up to 24 hours (Figure 2 and Table 5 & 6, See Additional files 5 &6). In fact at 48 hours after rhFSH injections, 10 out of 12 cases in abdominal injection group surpassed the FSH threshold while only 3 out of 12 cases in vaginal injection group had serum FSH concentration over the FSH threshold of 7.8 IU/L (Figure 4). This fact might explain a higher E2 level found around 48-72 hours in those of abdominal injections even though in general the rhFSH absorption was higher in those of vaginal injections. The level of E2 dropped sharply to baseline level after 96 hours reflects the serum concentrations of FSH may not have maintained for follicular growth. In contrast, E2 levels in those who received vaginal injections were maintained at above baseline level after 120 hours with a second peak at 240 hours and then dropped sharply to below baseline level thereafter. This pattern of E2 levels was well correlated with plateau concentrations of FSH in those who received vaginal injections. As this study represents only limited numbers of cases, the disparity of estradiol and FSH levels in the different administration modes, however, needs further investigation. Though follicle growth was not completely examined in the present study, our previous works showed proper folliculogenesis with mature oocyte numbers, fertilization rates, and pregnancy rates were achieved in the intermittent vaginal administration of rhFSH in IVF treatment [14]. The doses of rhFSH needed for ovulation stimulation in clinical IVF treatment were half of those used for conventional SC injection method [14]. Thus, intermittent vaginal injection may provide an alternative treatment regime, avoiding excessive use of gonadotrophins for controlled ovulation stimulation which may lower the clinical pregnancy and live birth rates [32].

There is a trend of reducing injections of gonadotropins in the last decade. The main advantages of less frequent dosing are an increase in patient convenience, fewer chances for mistakes during drug administration and improved compliance [33]. Our present work with enhanced extent of absorption and longer apparent plasma half-life of rhFSH provides the pharmacokinetic basis for the intermittent injections. Besides intermittent vaginal injections, there were few studies also aimed to reduce the injections of gonadotropins. Intermittent administration of rhFSH (every 3 days up to day 6) followed by daily injection, has been applied in IVF treatment and significantly reduces the total number of injections, compared with a conventional FSH regimen [34]. A loading dose of 300 IU rhFSH followed by 3 treatment free days, and a return to daily injections has also been successfully used for ovulation induction in polycystic ovarian syndrome [35]. Recent development of long acting FSH which was administered as single dose to sustain the FSH level for 7 days followed by daily injection of rhFSH also can achieve similar ovarian stimulating effects compared with a conventional FSH regimen [36].

Subcutaneous injection of FSH can cause some side reactions, depending on variables such as medicines, needle size and injected volume. In this study, injection of rhFSH via both abdominal and vaginal routes was well tolerated and local reactions were all mild and short-lived. No apparent differences with respect to local tolerance were observed. A detailed pre-injection counselling and description of the new method is mandatory to reassure the patients that the procedure is unlikely to cause significant discomfort. Generally, the method of administration by the vaginal route is more difficult than abdominal injections and requires a health professional to accomplish. However, the slight burden of physician/nurses provides advantages of less frequent dosing with an increase in patient convenience and fewer chances for mistakes during drug administration.

There are few shortcomings in the original design of this trial including (1) the small numbers of the subject enrolled which might result in less significant power in the statistical analysis; and (2) the endogenous FSH was not fully suppressed which made it difficult to provide a reliable pharmacokinetic analysis especially in the elimination half-life. As the volunteers were generally difficult to recruit in the cross-over study, the number of the subjects used in previous similar analysis of pharmacokinetics on rhFSH [15,17-20] was followed. In this two-period cross-over trial of 12 volunteers, the mean log C_max _for abdominal injection was 1.14 and that for vaginal injection was 1.24 and the pooled within-subject variation was SD = 0.10. If the two-sided power is set at 80%, with the test size 10%, then N = 6 to 8 subjects would need to be recruited in the bioequivalence of the ratio of two means [37,38]. This indicates that 12 cases are enough for this study. Future study with more subjects and with fully suppressed endogenous FSH may help to clarify those issues and provide even higher power in the analysis.

## Conclusion

We conclude that rhFSH administered vaginally has a higher rate and extent of absorption. Though the data in this study were for a single injection, the efficiency of intermittent serial vaginal injections of rhFSH had been analyzed in another study, which showed proper folliculogenesis and the in vitro fertilization rate and clinical pregnancy rate [14]. This injection mode thus provides a more cost-effective regimen for gonadotropin therapy. The potential therapeutic value of higher and prolonged FSH concentrations, as in vaginal administration, warrants further consideration.

## Competing interests

The authors declare that they have no competing interests.

## Authors' contributions

CCH participated in the design and carry on the study and drafting the manuscript. HCK contributed to statistical analysis of data and helped to draft the manuscript. CTH participated in its design and coordination and helped to draft the manuscript. QG contributed to conception of the study and acquisition of data. All authors read and approved the final manuscript.

## Supplementary Material

Additional file 1 **Pharmacokinetic parameters for abdominal subcutaneous injection (t = 120 hour).** Click here for file

Additional file 2 **Pharmacokinetic parameters for vaginal subcutaneous injection (t = 120 hour).** Click here for file

Additional file 3 **Statistical analysis for pharmacokinetic parameters of vaginal versus abdominal rhFSH injections.** Click here for file

Additional file 4 **Repeated measures on the tests of between-subjects and within-subjects effects.** Click here for file

Additional file 5 **Post hoc multiple comparisons of plasma FSH levels in abdominal injection using Dunnett t test.** Click here for file

Additional file 6 **Post hoc multiple comparisons of plasma FSH levels in vaginal injection using Dunnett t test.** Click here for file
