# Supplementary material for: The absorption and uptake of recombinant human follicle-stimulating hormone through vaginal subcutaneous injections - a pharmacokinetic study
Source: Reprod Biol Endocrinol. 2009 Oct 7;7:107. doi: 10.1186/1477-7827-7-107 (PMC2764710; doi:10.1186/1477-7827-7-107)
Supplement: Additional file 1 — Pharmacokinetic parameters for abdominal subcutaneous injection (t = 120 hour). [file 1477-7827-7-107-S1.DOC]

Table 1. Pharmacokinetic parameters for abdominal subcutaneous injection (t=120 hour)

| **Subjects** | **Area under the plasma concentration-time curve,** **AUC_0-t_** | **Area under the plasma concentration-time curve,** **AUC_0-∞_** | **AUC_0-t_/ AUC_0-∞_** | **Maximal plasma concentration,**  **C_max_** | **Time to reach measured maximal plasma concentration**, **T_max_** | **Mean residence time, MRT** | **Apparent half-life, t_1/2_** | **Plasma elimination rate constant, K_el_** | **Square of the** [**correlation coefficient**](http://en.wikipedia.org/wiki/Correlation_coefficient)**,**  **RSQ** | **Volume of distribution, V_z_** | **Total body clearance , Cl** |
| --- | --- | --- | --- | --- | --- | --- | --- | --- | --- | --- | --- |
|  | **(mIU·h mL^–1^)** | **(mIU·h mL^–1^)** | **(%)** | **(mIU mL^–1^)** | **(h)** | **(h)** | **(h)** | **(h^-1^)** |  | **(mL)** | **(mL h^–1^)** |
| a | 846.4 | 942.6 | 89.8 | 15.90 | 12.00 | 51.79 | 34.04 | 0.020 | 0.9398 | 24009.8 | 463.6 |
| b | 839.3 | 1006.6 | 83.4 | 13.60 | 6.00 | 67.90 | 42.94 | 0.016 | 0.9998 | 29479.4 | 434.1 |
| c | 1197.9 | 1626.1 | 73.7 | 12.70 | 24.00 | 98.67 | 70.00 | 0.010 | 0.7411 | 26515.6 | 268.7 |
| d | 829.3 | 961.8 | 86.2 | 10.60 | 12.00 | 65.09 | 35.88 | 0.019 | 0.9971 | 29572.2 | 454.3 |
| e | 1288.3 | 1524.0 | 84.5 | 16.20 | 24.00 | 67.12 | 42.11 | 0.016 | 0.9970 | 19245.9 | 286.8 |
| f | 889.0 | 1054.0 | 84.3 | 17.00 | 10.00 | 65.65 | 45.74 | 0.015 | 0.9868 | 27218.7 | 414.6 |
| g | 852.2 | 1009.2 | 84.4 | 12.00 | 12.00 | 65.11 | 41.55 | 0.017 | 0.9886 | 28191.6 | 433.0 |
| h | 785.5 | 909.6 | 86.4 | 12.80 | 2.00 | 62.84 | 37.09 | 0.019 | 0.9967 | 30190.6 | 480.4 |
| i | 787.0 | 1010.8 | 77.9 | 12.50 | 10.00 | 77.21 | 50.05 | 0.014 | 0.9578 | 33380.5 | 432.3 |
| j | 806.4 | 1036.7 | 77.8 | 12.20 | 12.00 | 80.42 | 63.84 | 0.011 | 0.9900 | 33901.9 | 421.5 |
| k | 886.3 | 1046.8 | 84.7 | 13.50 | 24.00 | 66.97 | 41.21 | 0.017 | 0.9998 | 27956.3 | 417.5 |
| l | 1153.0 | 1482.6 | 77.8 | 18.50 | 12.00 | 76.84 | 49.56 | 0.014 | 0.9616 | 22648.4 | 294.8 |
| Mean | 930.0 | 1134.2 | 82.6 | 13.96 | 13.33 | 70.47 | 46.17 | 0.016 | 0.9630 | 27692.6 | 400.1 |
| SD | 176.2 | 252.8 | 4.7 | 2.38 | 7.10 | 11.67 | 10.93 | 0.003 | 0.0726 | 4222.8 | 73.2 |
| CV | 18.9 | 22.3 | 5.7 | 17.0 | 53.3 | 16.6 | 23.7 | 20.2 | 7.5 | 15.2 | 18.3 |

All pharmacokinetic parameters were calculated by non-compartment methods using WinNonlin^TM^, version 5.2.

h: hour(s).
