# Supplementary material for: The absorption and uptake of recombinant human follicle-stimulating hormone through vaginal subcutaneous injections - a pharmacokinetic study
Source: Reprod Biol Endocrinol. 2009 Oct 7;7:107. doi: 10.1186/1477-7827-7-107 (PMC2764710; doi:10.1186/1477-7827-7-107)
Supplement: Additional file 2 — Pharmacokinetic parameters for vaginal subcutaneous injection (t = 120 hour). [file 1477-7827-7-107-S2.DOC]

Table 2. Pharmacokinetic parameters for vaginal subcutaneous injection (t=120 hour)

| **Subjects** | **Area under the plasma concentration-time curve,** **AUC_0-t_** | **Area under the plasma concentration-time curve,** **AUC_0-∞_** | **AUC_0-t_/ AUC_0-∞_** | **Maximal plasma concentration,**  **C_max_** | **Time to reach measured maximal plasma concentration**, **T_max_** | **Mean residence time, MRT** | **Apparent half-life, t_1/2_** | **Plasma elimination rate constant, K_el_** | **Square of the** [**correlation coefficient**](http://en.wikipedia.org/wiki/Correlation_coefficient)**,**  **RSQ** | **Volume of distribution, V_z_** | **Total body clearance , Cl** |
| --- | --- | --- | --- | --- | --- | --- | --- | --- | --- | --- | --- |
|  | **(mIU·h mL^–1^)** | **(mIU·h mL^–1^)** | **(%)** | **(mIU mL^–1^)** | **(h)** | **(h)** | **(h)** | **(h^-1^)** |  | **(mL)** | **(mL h^–1^)** |
| a | 742.4 | 983.5 | 75.5 | 12.80 | 14.00 | 79.22 | 49.29 | 0.014 | 0.8643 | 35201.6 | 444.3 |
| b | 925.7 | 1393.7 | 66.4 | 17.40 | 2.00 | 99.69 | 60.07 | 0.012 | 0.8730 | 31259.3 | 313.6 |
| c | 1491.8 | 2062.5 | 72.3 | 29.60 | 2.00 | 86.69 | 54.04 | 0.013 | 0.9171 | 18367.9 | 211.9 |
| d | 1663.1 | 3136.1 | 53.0 | 22.00 | 8.00 | 149.66 | 97.24 | 0.007 | 0.9050 | 20853.5 | 139.3 |
| e | 855.0 | 1382.1 | 61.9 | 18.50 | 6.00 | 115.07 | 71.93 | 0.010 | 0.8224 | 36382.2 | 316.2 |
| f | 1249.5 | 1942.0 | 64.3 | 19.65 | 8.00 | 107.50 | 66.48 | 0.010 | 0.8546 | 24189.1 | 225.0 |
| g | 909.3 | 1437.6 | 63.3 | 13.70 | 4.00 | 112.99 | 73.53 | 0.009 | 0.8165 | 34346.7 | 304.0 |
| h | 931.9 | 1452.5 | 64.2 | 15.70 | 6.00 | 107.87 | 66.58 | 0.010 | 0.8429 | 32454.8 | 300.9 |
| i | 755.6 | 1021.8 | 73.9 | 12.50 | 8.00 | 79.68 | 47.56 | 0.015 | 0.8423 | 34077.6 | 427.7 |
| j | 1048.9 | 2000.3 | 52.4 | 18.30 | 6.00 | 142.95 | 86.31 | 0.008 | 0.6718 | 31230.5 | 218.5 |
| k | 887.1 | 1316.9 | 67.4 | 15.89 | 6.00 | 93.43 | 53.97 | 0.013 | 0.7566 | 25837.3 | 331.8 |
| l | 1010.7 | 1553.3 | 65.1 | 17.25 | 10.00 | 104.2 | 63.96 | 0.011 | 0.8379 | 25247.7 | 273.6 |
| Mean | 1039.3 | 1640.2 | 65.0 | 17.77 | 6.67 | 106.58 | 65.91 | 0.011 | 0.8337 | 29120.7 | 292.2 |
| SD | 287.0 | 585.1 | 7.2 | 4.67 | 3.34 | 22.13 | 14.86 | 0.002 | 0.0659 | 5998.5 | 87.6 |
| CV | 27.6 | 35.7 | 11.0 | 26.2 | 50.1 | 20.8 | 22.5 | 20.9 | 7.9 | 20.6 | 30.0 |

All pharmacokinetic parameters were calculated by non-compartment methods using WinNonlin^TM^, version 5.2. h: hour(s).
