# Supplementary material for: The absorption and uptake of recombinant human follicle-stimulating hormone through vaginal subcutaneous injections - a pharmacokinetic study
Source: Reprod Biol Endocrinol. 2009 Oct 7;7:107. doi: 10.1186/1477-7827-7-107 (PMC2764710; doi:10.1186/1477-7827-7-107)
Supplement: Additional file 3 — Statistical analysis for pharmacokinetic parameters of vaginal versus abdominal rhFSH injections. [file 1477-7827-7-107-S3.DOC]

Table 3. Statistical analysis for pharmacokinetic parameters of vaginal versus abdominal rhFSH injections

| **Parameters** | **Abdominal sc injection (n=12)** | | | |  | **Vaginal sc injection (n=12)** | | | | **P-value** |
| --- | --- | --- | --- | --- | --- | --- | --- | --- | --- | --- |
|  | **Mean** |  | **SD** | **95% confidence interval** |  | **Mean** |  | **SD** | **95% confidence interval** |  |
| **AUC_0-t_ (mIU·h mL^–1^)** | 930.0 | ± | 176.2 | (818.1, 1042.0) |  | 1039.3 | ± | 287.0 | (856.9, 1221.6) | 0.266 |
| **AUC_0-∞_ (mIU·h mL^–1^)** | 1134.2 | ± | 252.8 | (937.6, 1294.8) |  | 1640.0 | ± | 585.1 | (1268.4, 2012.0) | 0.021 |
| **C_max_ (mIU mL^–1^)** | 13.96 | ± | 2.38 | (12.45, 15.47) |  | 17.77 | ± | 4.67 | (14.81, 20.74) | 0.035 |
| **t_max_ (h)** | 13.33 | ± | 7.10 | (8.82, 17.85) |  | 6.67 | ± | 3.34 | (4.54, 8.79) | 0.023 |
| **MRT (h)** | 70.47 | ± | 11.67 | (63.05, 77.88) |  | 106.58 | ± | 22.13 | (92.52, 120.64) | < 0.001 |
| **t_1/2_ (h)** | 46.17 | ± | 10.93 | (39.22, 53.11) |  | 65.91 | ± | 14.86 | (56.48, 75.35) | 0.006 |
| **K_el_ (h^-1^)** | 0.016 | ± | 0.003 | (0.014, 0.018) |  | 0.011 | ± | 0.002 | (0.009, 0.013) | 0.004 |
| **V_z_ (mL)** | 27692.6 | ± | 4222.8 | (25009.5, 30375.6) |  | 29120.7 | ± | 5998.5 | (25309.4, 32931.9) | 0.541 |
| **CL (mL h^–1^)** | 400.1 | ± | 73.2 | (353.7, 446.6) |  | 292.2 | ± | 87.6 | (236.6, 347.9) | 0.005 |

P values: one way ANOVA.

h: hour(s).
