# Supplementary material for: The absorption and uptake of recombinant human follicle-stimulating hormone through vaginal subcutaneous injections - a pharmacokinetic study
Source: Reprod Biol Endocrinol. 2009 Oct 7;7:107. doi: 10.1186/1477-7827-7-107 (PMC2764710; doi:10.1186/1477-7827-7-107)
Supplement: Additional file 4 — Repeated measures on the tests of between-subjects and within-subjects effects. [file 1477-7827-7-107-S4.DOC]

Table 4. Repeated measures on the tests of between-subjects and within-subjects effects

| Source | Sum of Squares | Degree of  freedom | Mean Square | F value | P value |
| --- | --- | --- | --- | --- | --- |
| Tests of between-subjects effects | | | | | |
| Approach ^a^ | 605.74 | 1 | 605.74 | 8.85 ^*^ | 0.008^**^ |
| Error | 1231.84 | 18 | 68.44 |  |  |
| Tests of within-subjects effects | | | | | |
| Time ^b^ | 3378.35 | 12 | 281.53 | 76.15^**^ | 0.000^**^ |
| Time × Approach ^c^ | 282.04 | 12 | 23.50 | 6.36^**^ | 0.000^**^ |
| Error | 798.55 | 216 | 3.70 |  |  |

^a^ “Approach is significant” means that there are different effects between vaginal sc injection and abdominal sc injection.

^b^  “Time is significant” means that each approach has the different effects over time.

^c^ “Time × Approach is significant” means that time and approach have an interaction.

^**^P<0.01,
