# Supplementary material for: The absorption and uptake of recombinant human follicle-stimulating hormone through vaginal subcutaneous injections - a pharmacokinetic study
Source: Reprod Biol Endocrinol. 2009 Oct 7;7:107. doi: 10.1186/1477-7827-7-107 (PMC2764710; doi:10.1186/1477-7827-7-107)
Supplement: Additional file 5 — Post hoc multiple comparisons of plasma FSH levels in abdominal injection using Dunnett t test. [file 1477-7827-7-107-S5.DOC]

Table 5. Post hoc multiple comparisons of plasma FSH levels in abdominal injection using Dunnett t test.

| ***I*** Time (hours) | ***J*** Time (hours) | ***I-J*** Difference (IU/L) (mean ± s.e.) | P value | 95% Confidence interval | |
| --- | --- | --- | --- | --- | --- |
|  |  |  |  | Lower bound | Upper bound |
|  | | | | | |
| 1 | 0 | 3.24 ± 0.76 | 0.00^**^ | 0.99 | 5.50 |
| 2 | 0 | 3.89 ± 0.73 | 0.00 ^**^ | 1.73 | 6.04 |
| 4 | 0 | 5.04 ± 0.76 | 0.00 ^**^ | 2.79 | 7.30 |
| 6 | 0 | 6.95 ± 0.73 | 0.00 ^**^ | 4.80 | 9.10 |
| 8 | 0 | 7.39 ± 0.76 | 0.00 ^**^ | 5.13 | 9.65 |
| 10 | 0 | 7.86 ± 0.76 | 0.00 ^**^ | 5.60 | 10.11 |
| 12 | 0 | 7.35 ± 0.74 | 0.00 ^**^ | 5.15 | 9.55 |
| 24 | 0 | 7.23 ± 0.73 | 0.00 ^**^ | 5.07 | 9.38 |
| 48 | 0 | 4.26 ± 0.73 | 0.00 ^**^ | 2.10 | 6.41 |
| 72 | 0 | 1.05 ± 0.73 | 0.84 | -1.10 | 3.20 |
| 96 | 0 | -0.47 ± 0.73 | 1.00 | -2.62 | 1.68 |
| 120 | 0 | -1.94 ± 0.73 | 0.11 | -4.09 | 0.21 |
| 144 | 0 | -2.52 ± 1.36 | 0.54 | -6.55 | 1.50 |
| 192 | 0 | -2.47 ± 1.36 | 0.57 | -6.50 | 1.56 |
| 240 | 0 | -2.71 ± 1.36 | 0.44 | -6.74 | 1.31 |
| 288 | 0 | -2.79 ± 1.36 | 0.40 | -6.82 | 1.24 |
| 360 | 0 | -2.72 ± 1.36 | 0.43 | -6.75 | 1.30 |
|  | | | | | |

“*I* Time” indicates the time interval after the rhFSH administration and“*J* Time” indicates the baseline time point. I-J difference represents the difference of plasma FSH concentrations expressed with mean ± standard error.

^**^ P<0.01
