# Supplementary material for: The absorption and uptake of recombinant human follicle-stimulating hormone through vaginal subcutaneous injections - a pharmacokinetic study
Source: Reprod Biol Endocrinol. 2009 Oct 7;7:107. doi: 10.1186/1477-7827-7-107 (PMC2764710; doi:10.1186/1477-7827-7-107)
Supplement: Additional file 6 — Post hoc multiple comparisons of plasma FSH levels in vaginal injection using Dunnett t test. [file 1477-7827-7-107-S6.DOC]

Table 6. Post hoc multiple comparisons of plasma FSH levels in vaginal injection using Dunnett t test.

| ***I*** Time (hours) | ***J*** Time (hours) | ***I-J*** Difference (IU/L) (mean ± s.e.) | P value | 95% Confidence interval | |
| --- | --- | --- | --- | --- | --- |
|  |  |  |  | Lower bound | Upper bound |
|  | | | | | |
| 1 | 0 | 7.80 ± 0.85 | 0.00^**^ | 5.24 | 10.36 |
| 2 | 0 | 9.30 ± 0.83 | 0.00^**^ | 6.79 | 11.80 |
| 4 | 0 | 9.54 ± 0.85 | 0.00^**^ | 6.98 | 12.10 |
| 6 | 0 | 10.90 ± 0.83 | 0.00^**^ | 8.39 | 13.40 |
| 8 | 0 | 10.97 ± 0.85 | 0.00^**^ | 8.41 | 13.53 |
| 10 | 0 | 10.44 ± 0.85 | 0.00^**^ | 7.88 | 13.00 |
| 12 | 0 | 10.05 ± 0.83 | 0.00^**^ | 7.55 | 12.56 |
| 24 | 0 | 7.75 ± 0.83 | 0.00^**^ | 5.25 | 10.25 |
| 48 | 0 | 2.47 ± 0.83 | 0.06 | -0.03 | 4.97 |
| 72 | 0 | 0.59 ± 0.83 | 1.00 | -1.91 | 3.09 |
| 96 | 0 | 0.43 ± 0.83 | 1.00 | -2.07 | 2.93 |
| 120 | 0 | 0.80 ± 0.83 | 1.00 | -1.71 | 3.30 |
| 144 | 0 | -0.55 ± 1.18 | 1.00 | -4.09 | 2.99 |
| 192 | 0 | -1.07 ± 1.18 | 1.00 | -4.61 | 2.47 |
| 240 | 0 | 2.42 ± 1.18 | 0.44 | -1.12 | 5.96 |
| 288 | 0 | -0.19 ± 1.09 | 1.00 | -3.45 | 3.08 |
| 360 | 0 | 0.23 ± 1.09 | 1.00 | -3.03 | 3.50 |
|  | | | | | |

Footnotes as those labelled for Table 5.
